# Supplementary material for: Developing an in silico minimum inhibitory concentration panel test for Klebsiella pneumoniae
Source: Sci Rep. 2018 Jan 11;8:421. doi: 10.1038/s41598-017-18972-w (PMC5765115; doi:10.1038/s41598-017-18972-w)
Supplement: Supplementary file 1 — Supplementary Information [file 41598_2017_18972_MOESM1_ESM.pdf]

Developing an *in silico* minimum inhibitory concentration panel test for *Klebsiella pneumoniae* Supplemental Information

Marcus Nguyen[1,2,3], Thomas Brettin[2,3], S. Wesley Long[4,5], James M. Musser[4,5], Randall J. Olsen[4,5], Robert Olson[2,3], Maulik Shukla[2,3], Rick L. Stevens[2,3,7], Fangfang Xia[2,3], Hyunseung Yoo[2,3], James J. Davis[2,3,*]

[1]Northern Illinois University, Computation Science, DeKalb, IL, 60115, USA
[2]University of Chicago, Computation Institute, Chicago, IL, 60637, USA
[3]Argonne National Laboratory, Computing Environment and Life Sciences, Argonne, IL, 60439, USA
[4]Center for Molecular and Translational Human Infectious Diseases Research, Department of Pathology and Genomic Medicine, Houston Methodist Research Institute and Houston Methodist Hospital, Houston, Texas, 77030, USA
[5]Department of Pathology and Laboratory Medicine, Weill Cornell Medical College, New York, New York, 10065, USA
[7]University of Chicago, Department of Computer Science, Chicago, IL, 60439, USA
[*]corresponding author jjdavis@anl.gov

Table S1. A comparison of raw accuracies and accuracies within ±1 two-fold dilution step of the actual MIC for the XGBoost model

Antibiotic	Samples	Raw Accuracy[a]	Raw Accuracy[a] 95% C.I.[b]	Within 1 two-fold Accuracy[c]	Within 1 two-fold[b] 95% C.I.[b]
All	32705	0.69	0.68, 0.69	0.92	0.92, 0.92
Amikacin	1667	0.69	0.67, 0.71	0.97	0.96, 0.98
Ampicillin	1666	0.95	0.94, 0.96	1.00	0.99, 1.00
Ampicillin/Sulbactam	1664	0.88	0.86, 0.90	0.99	0.99, 1.00
Aztreonam	1644	0.62	0.6, 0.65	0.89	0.89, 0.90
Cefazolin	1667	0.85	0.84, 0.86	0.96	0.95, 0.96
Cefepime	1571	0.19	0.16, 0.22	0.61	0.58, 0.64
Cefoxitin	1645	0.40	0.37, 0.43	0.90	0.89, 0.91
Ceftazidime	1667	0.73	0.71, 0.76	0.92	0.91, 0.93
Ceftriaxone	1667	0.73	0.71, 0.75	0.89	0.87, 0.90
Cefuroxime sodium	1575	0.94	0.93, 0.95	0.99	0.99, 1.00
Ciprofloxacin	1664	0.87	0.85, 0.89	0.98	0.97, 0.98
Gentamicin	1667	0.65	0.62, 0.68	0.95	0.93, 0.96
Imipenem	1666	0.70	0.68, 0.71	0.94	0.93, 0.95
Levofloxacin	1666	0.82	0.80, 0.83	0.97	0.96, 0.97
Meropenem	1660	0.69	0.66, 0.71	0.93	0.91, 0.95
Nitrofurantoin	895	0.78	0.75, 0.82	0.96	0.95, 0.97
Piperacillin/Tazobactam	1662	0.46	0.43, 0.49	0.78	0.77, 0.79
Tetracycline	1667	0.50	0.48, 0.53	0.89	0.87, 0.90
Tobramycin	1666	0.61	0.57, 0.64	0.95	0.94, 0.96
Trimethoprim/Sulfamethoxazole	1667	0.74	0.72, 0.76	0.95	0.94, 0.96

[a] Average raw accuracy; raw accuracy is defined as predicting the actual MIC.
[b] 95% confidence interval.
[c] Average within ±1 two-fold dilution accuracy.

Table S2. The within 1-tier accuracies for all antibiotic-MIC combinations.

Antibiotic	MIC	Samples	Accuracy[a]	95% C.I.[b]
All	0.02	1	0.00	*nan*, *nan*
All	0.03	1	0.00	*nan*, *nan*
All	0.09	1	0.00	*nan*, *nan*
All	0.13	282	0.92	0.86, 0.97
All	0.25	120	0.91	0.85, 0.97
All	0.38	1	0.00	*nan*, *nan*
All	0.5	784	0.72	0.69, 0.75
All	1.0	3118	0.84	0.83, 0.85
All	2.0	1775	0.80	0.78, 0.81
All	4.0	4459	0.93	0.92, 0.94
All	6.0	2	0.00	0.00, 0.00
All	8.0	3737	0.95	0.95, 0.96
All	11.0	2	1.00	1.00, 1.00
All	12.0	2	0.50	−5.85, 6.85
All	14.0	2	0.50	−5.85, 6.85
All	16.0	3896	0.92	0.91, 0.92
All	17.0	4	1.00	1.00, 1.00
All	18.0	11	0.65	0.31, 0.99
All	19.0	12	1.00	1.00, 1.00
All	20.0	15	1.00	1.00, 1.00
All	21.0	4	1.00	1.00, 1.00
All	23.0	1	1.00	*nan*, *nan*
All	32.0	10802	0.96	0.96, 0.96
All	50.0	5	0.20	−0.36, 0.76
All	64.0	1834	0.94	0.93, 0.96
All	128.0	1833	0.91	0.89, 0.93
All	512.0	1	0.00	*nan*, *nan*
Amikacin	4.0	155	0.94	0.89, 0.99
Amikacin	8.0	1058	0.99	0.99, 1.00
Amikacin	16.0	107	1.00	1.00, 1.00
Amikacin	32.0	244	0.98	0.96, 1.00
Amikacin	64.0	103	0.70	0.64, 0.76
Ampicillin	2.0	1	0.00	*nan*, *nan*
Ampicillin	4.0	1	0.00	*nan*, *nan*
Ampicillin	8.0	2	1.00	1.00, 1.00
Ampicillin	16.0	27	1.00	1.00, 1.00
Ampicillin	32.0	1635	1.00	1.00, 1.00
Ampicillin/Sulbactam	2.0	3	0.33	−1.10, 1.77
Ampicillin/Sulbactam	4.0	10	0.90	0.67, 1.13
Ampicillin/Sulbactam	8.0	77	0.96	0.90, 1.02
Ampicillin/Sulbactam	16.0	119	1.00	1.00, 1.00
Ampicillin/Sulbactam	32.0	1455	1.00	0.99, 1.00

Antibiotic	MIC	Samples	Accuracy[a]	95% C.I.[b]
Aztreonam	1.0	81	0.79	0.69, 0.88
Aztreonam	2.0	99	0.12	0.04, 0.20
Aztreonam	4.0	36	0.08	−0.01, 0.18
Aztreonam	8.0	21	0.67	0.43, 0.91
Aztreonam	16.0	92	1.00	1.00, 1.00
Aztreonam	32.0	1315	0.98	0.97, 0.98
Cefazolin	1.0	42	0.24	0.10, 0.39
Cefazolin	2.0	26	0.73	0.49, 0.97
Cefazolin	4.0	11	0.25	−0.05, 0.55
Cefazolin	8.0	7	0.86	0.51, 1.21
Cefazolin	16.0	11	0.65	0.31, 0.99
Cefazolin	32.0	1478	1.00	1.00, 1.00
Cefazolin	64.0	92	0.80	0.67, 0.94
Cefepime	0.5	84	0.84	0.76, 0.92
Cefepime	1.0	307	0.08	0.05, 0.11
Cefepime	2.0	27	0.50	0.22, 0.78
Cefepime	4.0	119	0.84	0.78, 0.90
Cefepime	8.0	71	0.97	0.93, 1.01
Cefepime	16.0	173	0.94	0.89, 0.99
Cefepime	32.0	790	0.65	0.61, 0.70
Cefoxitin	4.0	423	0.80	0.77, 0.83
Cefoxitin	8.0	244	0.97	0.95, 1.00
Cefoxitin	16.0	150	0.97	0.94, 0.99
Cefoxitin	32.0	828	0.92	0.91, 0.94
Ceftazidime	0.5	86	0.40	0.28, 0.51
Ceftazidime	1.0	28	0.03	−0.04, 0.11
Ceftazidime	2.0	10	0.30	−0.05, 0.65
Ceftazidime	4.0	12	0.65	0.31, 0.99
Ceftazidime	8.0	43	0.84	0.73, 0.95
Ceftazidime	16.0	103	0.97	0.94, 1.00
Ceftazidime	32.0	1385	0.97	0.96, 0.98
Ceftriaxone	0.5	80	0.17	0.08, 0.27
Ceftriaxone	2.0	59	0.12	0.04, 0.20
Ceftriaxone	4.0	9	0.56	0.15, 0.96
Ceftriaxone	8.0	5	0.40	−0.28, 1.08
Ceftriaxone	16.0	29	0.82	0.63, 1.00
Ceftriaxone	32.0	53	0.85	0.75, 0.96
Ceftriaxone	64.0	1432	0.96	0.96, 0.97
Cefuroxime sodium	4.0	75	0.91	0.86, 0.95
Cefuroxime sodium	8.0	16	0.95	0.84, 1.06
Cefuroxime sodium	16.0	15	0.95	0.84, 1.06
Cefuroxime sodium	32.0	1469	1.00	1.00, 1.00
Ciprofloxacin	0.5	158	0.80	0.73, 0.86
Ciprofloxacin	1.0	43	0.97	0.92, 1.03
Ciprofloxacin	2.0	39	1.00	1.00, 1.00
Ciprofloxacin	4.0	1424	1.00	0.99, 1.00

Antibiotic	MIC	Samples	Accuracy[a]	95% C.I.[b]
Gentamicin	0.5	13	0.65	0.36, 0.94
Gentamicin	1.0	94	0.90	0.85, 0.94
Gentamicin	2.0	645	0.97	0.95, 0.98
Gentamicin	4.0	174	0.99	0.97, 1.01
Gentamicin	8.0	58	0.91	0.85, 0.98
Gentamicin	16.0	683	0.93	0.92, 0.94
Imipenem	0.25	111	0.90	0.83, 0.97
Imipenem	0.5	38	0.94	0.85, 1.03
Imipenem	1.0	1011	0.96	0.95, 0.98
Imipenem	2.0	28	0.60	0.38, 0.82
Imipenem	4.0	37	0.84	0.69, 1.00
Imipenem	6.0	1	0.00	*nan*, *nan*
Imipenem	8.0	107	0.94	0.87, 1.02
Imipenem	16.0	333	0.92	0.88, 0.95
Levofloxacin	1.0	331	0.90	0.87, 0.93
Levofloxacin	2.0	18	0.80	0.55, 1.05
Levofloxacin	4.0	30	1.00	1.00, 1.00
Levofloxacin	8.0	1287	0.98	0.97, 0.99
Meropenem	0.02	1	0.00	*nan*, *nan*
Meropenem	0.03	1	0.00	*nan*, *nan*
Meropenem	0.09	1	0.00	*nan*, *nan*
Meropenem	0.13	147	0.92	0.86, 0.98
Meropenem	0.25	1	1.00	*nan*, *nan*
Meropenem	0.38	1	0.00	*nan*, *nan*
Meropenem	1.0	982	0.97	0.96, 0.98
Meropenem	2.0	45	0.60	0.50, 0.71
Meropenem	4.0	64	0.78	0.65, 0.90
Meropenem	6.0	1	0.00	*nan*, *nan*
Meropenem	8.0	50	0.92	0.85, 0.99
Meropenem	12.0	2	0.50	−5.85, 6.85
Meropenem	16.0	364	0.90	0.85, 0.95
Nitrofurantoin	16.0	17	0.25	−0.00, 0.50
Nitrofurantoin	32.0	38	0.79	0.65, 0.94
Nitrofurantoin	64.0	121	1.00	1.00, 1.00
Nitrofurantoin	128.0	719	0.98	0.97, 0.99
Piperacillin/Tazobactam	2.0	25	0.45	0.37, 0.53
Piperacillin/Tazobactam	4.0	130	0.45	0.41, 0.50
Piperacillin/Tazobactam	8.0	117	0.28	0.18, 0.39
Piperacillin/Tazobactam	16.0	160	0.68	0.59, 0.77
Piperacillin/Tazobactam	32.0	106	0.97	0.94, 1.00
Piperacillin/Tazobactam	64.0	76	0.96	0.90, 1.02
Piperacillin/Tazobactam	128.0	1048	0.87	0.84, 0.89
Tetracycline	1.0	51	0.82	0.78, 0.87
Tetracycline	2.0	214	0.61	0.54, 0.69
Tetracycline	4.0	474	0.99	0.98, 1.00
Tetracycline	8.0	150	0.99	0.98, 1.01
Tetracycline	16.0	778	0.89	0.87, 0.90

**Antibiotic**	**MIC**	**Samples**	**Accuracy**[a]	**95% C.I.**[b]
Tobramycin	0.5	4	0.00	0.00, 0.00
Tobramycin	1.0	91	0.91	0.86, 0.96
Tobramycin	2.0	471	0.93	0.90, 0.96
Tobramycin	4.0	23	0.90	0.75, 1.05
Tobramycin	8.0	354	0.99	0.97, 1.00
Tobramycin	16.0	723	0.96	0.94, 0.98
Trimethoprim/Sulfamethoxazole	0.5	316	0.85	0.80, 0.91
Trimethoprim/Sulfamethoxazole	1.0	54	0.83	0.69, 0.97
Trimethoprim/Sulfamethoxazole	2.0	46	0.98	0.93, 1.03
Trimethoprim/Sulfamethoxazole	4.0	1251	0.98	0.97, 0.99

[a] Average within ± 1 two-fold dilution accuracy.
[b] 95% confidence interval.

Table S4. The top three PATRIC functions that are most highly correlated with the laboratory tested MICs for each antibiotic, and the corresponding PCCs based on MICs that are predicted by the model.

Antibiotic	PATRIC Function	PCC Actual MIC	PCC Predicted MIC
Amikacin	IncI1 plasmid conjugative transfer prepilin PilS	0.577	0.478
	type III restriction enzyme, res subunit	0.573	0.025
	integral membrane protein	0.573	0.025
Ampicillin	Class A beta-lactamase (EC 3.5.2.6) => TEM family	0.357	0.327
	Integron integrase IntI1	0.351	0.309
	Class A beta-lactamase (EC 3.5.2.6) => CTX-M family, extended-spectrum	0.340	0.309
Ampicillin_Sulbactam	Class A beta-lactamase (EC 3.5.2.6) => TEM family	0.780	0.787
	Class A beta-lactamase (EC 3.5.2.6) => CTX-M family, extended-spectrum	0.772	0.764
	Tryptophan synthase (indole-salvaging) (EC 4.2.1.122)	0.752	0.774
Aztreonam	Integron integrase IntI1	0.678	0.614
	Class A beta-lactamase (EC 3.5.2.6) => TEM family	0.659	0.752
	Tryptophan synthase (indole-salvaging) (EC 4.2.1.122)	0.637	0.627
Cefazolin	Class A beta-lactamase (EC 3.5.2.6) => CTX-M family, extended-spectrum	0.676	0.667
	Integron integrase IntI1	0.673	0.663
	Tryptophan synthase (indole-salvaging) (EC 4.2.1.122)	0.659	0.678
Cefepime	Class A beta-lactamase (EC 3.5.2.6) => CTX-M family, extended-spectrum	0.848	0.648
	Tryptophan synthase (indole-salvaging) (EC 4.2.1.122)	0.833	0.666
	Class A beta-lactamase (EC 3.5.2.6) => TEM family	0.742	0.691
Cefoxitin	Class A beta-lactamase (EC 3.5.2.6) => KPC family, carbapenem-hydrolyzing	0.550	0.571
	DUF3701 domain / Site-specific tyrosine recombinase	0.502	0.561
	H repeat-associated protein, YhhI family	0.451	0.554
Ceftazidime	Integron integrase IntI1	0.657	0.623
	Class A beta-lactamase (EC 3.5.2.6) => TEM family	0.655	0.763
	Tryptophan synthase (indole-salvaging) (EC 4.2.1.122)	0.648	0.686
Ceftriaxone	Class A beta-lactamase (EC 3.5.2.6) => CTX-M family, extended-spectrum	0.823	0.700
	Tryptophan synthase (indole-salvaging) (EC 4.2.1.122)	0.802	0.714
	Class A beta-lactamase (EC 3.5.2.6) => TEM family	0.769	0.797

Antibiotic	PATRIC Function	PCC Actual MIC	PCC Predicted MIC
Cefuroxime sodium	Aminoglycoside N(3)-acetyltransferase (EC 2.3.1.81) => AAC(3)-II,III,IV,VI,VIII,IX,X	0.668	0.616
	FIG00639751: hypothetical protein	0.527	-0.060
	FIG00642442: hypothetical protein	0.527	-0.060
Ciprofloxacin	Integron integrase IntI1	0.715	0.713
	Tryptophan synthase (indole-salvaging) (EC 4.2.1.122)	0.692	0.758
	Class A beta-lactamase (EC 3.5.2.6) => TEM family	0.681	0.782
Gentamicin	Aminoglycoside N(3)-acetyltransferase (EC 2.3.1.81) => AAC(3)-II,III,IV,VI,VIII,IX,X	0.818	0.862
	FIG00732118: hypothetical protein	0.577	0.551
	Class A beta-lactamase (EC 3.5.2.6) => TEM family	0.571	0.637
Imipenem	Class A beta-lactamase (EC 3.5.2.6) => KPC family, carbapenem-hydrolyzing	0.891	0.905
	DUF3701 domain / Site-specific tyrosine recombinase	0.828	0.865
	H repeat-associated protein, YhhI family	0.761	0.853
Levofloxacin	probable bacteriophage protein STY1063	0.588	0.584
	DNA helicase (EC 3.6.4.12), phage-associated	0.568	0.602
	colicin immunity protein	0.557	0.571
Meropenem	Class A beta-lactamase (EC 3.5.2.6) => KPC family, carbapenem-hydrolyzing	0.923	0.814
	DUF3701 domain / Site-specific tyrosine recombinase	0.832	0.799
	H repeat-associated protein, YhhI family	0.733	0.818
Nitrofurantoin	Integron integrase IntI1	0.433	0.507
	Class A beta-lactamase (EC 3.5.2.6) => TEM family	0.423	0.491
	TonB protein	0.400	0.413
Piperacillin_Tazobactam	plasmid stabilization system	0.583	0.501
	Class A beta-lactamase (EC 3.5.2.6) => TEM family	0.581	0.662
	probable bacteriophage protein STY1063	0.487	0.592
Tetracycline	Tetracycline resistance regulatory protein TetR	0.829	0.717
	Tetracycline resistance, MFS efflux pump => Tet(A)	0.628	0.851
	Dihydropteroate synthase type-2 (EC 2.5.1.15) @ Sulfonamide resistance protein	0.530	0.657

Antibiotic	PATRIC Function	PCC Actual MIC	PCC Predicted MIC
Tobramycin	Aminoglycoside N(6')-acetyltransferase (EC 2.3.1.82) => AAC(6')-Ib/AAC(6')-II	0.837	0.853
	Class D beta-lactamase (EC 3.5.2.6) => OXA-1 family	0.685	0.704
	Chloramphenicol O-acetyltransferase (EC 2.3.1.28) => CatB family	0.654	0.703
Trimethoprim_Sulfamethoxazole	Dihydropteroate synthase type-2 (EC 2.5.1.15) @ Sulfonamide resistance protein	0.919	0.758
	Integron integrase IntI1	0.796	0.784
	Class A beta-lactamase (EC 3.5.2.6) => CTX-M family, extended-spectrum	0.705	0.773

Table S5. The within ±1-tier accuracy of the XGBoost model built from whole genome contigs, AMR genes only, and non-AMR genes respectively.

Antibiotic	Samples	Contigs Accuracy	95% C.I.	AMR Genes Accuracy	95% C.I.	Non-AMR Genes Accuracy	95% C.I.
All	32705	0.92	0.92, 0.92	0.92	0.91, 0.92	0.92	0.92, 0.92
Amikacin	1667	0.97	0.96, 0.98	0.96	0.95, 0.97	0.97	0.97, 0.98
Ampicillin	1666	1.00	0.99, 1.00	1.00	0.99, 1.00	1.00	0.99, 1.00
Ampicillin/Sulbactam	1664	0.99	0.99, 1.00	0.99	0.99, 1.00	0.99	0.99, 1.00
Aztreonam	1644	0.89	0.89, 0.90	0.89	0.88, 0.90	0.89	0.89, 0.90
Cefazolin	1667	0.96	0.95, 0.96	0.95	0.95, 0.96	0.96	0.96, 0.97
Cefepime	1571	0.61	0.58, 0.64	0.61	0.58, 0.63	0.60	0.58, 0.63
Cefoxitin	1645	0.90	0.89, 0.91	0.89	0.87, 0.90	0.90	0.89, 0.92
Ceftazidime	1667	0.92	0.91, 0.93	0.91	0.91, 0.92	0.91	0.90, 0.92
Ceftriaxone	1667	0.89	0.87, 0.90	0.90	0.89, 0.91	0.89	0.88, 0.91
Cefuroxime sodium	1575	0.99	0.99, 1.00	0.99	0.99, 1.00	0.99	0.99, 1.00
Ciprofloxacin	1664	0.98	0.97, 0.98	0.98	0.97, 0.98	0.97	0.97, 0.98
Gentamicin	1667	0.95	0.93, 0.96	0.96	0.95, 0.96	0.95	0.94, 0.96
Imipenem	1666	0.94	0.93, 0.95	0.92	0.91, 0.93	0.94	0.93, 0.95
Levofloxacin	1666	0.97	0.96, 0.97	0.97	0.96, 0.98	0.96	0.95, 0.97
Meropenem	1660	0.93	0.91, 0.95	0.91	0.89, 0.92	0.93	0.92, 0.94
Nitrofurantoin	895	0.96	0.95, 0.97	0.96	0.95, 0.96	0.96	0.96, 0.97
Piperacillin/Tazobactam	1662	0.78	0.77, 0.79	0.77	0.75, 0.80	0.77	0.76, 0.79
Tetracycline	1667	0.89	0.87, 0.90	0.90	0.89, 0.92	0.89	0.88, 0.90
Tobramycin	1666	0.95	0.94, 0.96	0.95	0.94, 0.96	0.93	0.92, 0.95
Trimethoprim/Sulfamethoxazole	1667	0.95	0.94, 0.96	0.94	0.93, 0.95	0.95	0.93, 0.96

Table S6. The number of different MIC values observed within the top five *K. pneumoniae* MLST types in the dataset.

Antibiotic	MLST type				
	307 (560 genomes)	258 (404 genomes)	16 (86 genomes)	15 (56 genomes)	280 (27 genomes)
Amikacin	5	6	3	4	2
Ampicillin	1	1	1	1	1
Ampicillin/Sulbactam	2	3	3	2	1
Aztreonam	5	5	5	4	2
Cefazolin	2	3	2	4	1
Cefepime	6	8	6	6	3
Cefoxitin	4	4	4	4	4
Ceftazidime	5	3	4	5	3
Ceftriaxone	3	6	3	3	1
Cefuroxime sodium	1	2	2	2	1
Ciprofloxacin	1	1	1	1	2
Gentamicin	6	6	5	4	1
Imipenem	8	6	3	6	1
Levofloxacin	3	2	2	2	3
Meropenem	7	10	4	3	1
Nitrofurantoin	4	1	3	4	3
Piperacillin/Tazobactam	7	7	7	7	5
Tetracycline	5	6	4	5	1
Tobramycin	7	5	4	4	2
Trimethoprim/Sulfamethoxazole	5	5	3	4	1

Table S7. The occurrence of AMR functions within the *K. pneumoniae* genomes. The column numbers correspond to the columns of the heatmap in in Figure S3 and the counts are the number of genomes with that AMR function.

Column	Count	Function
1	4	Class A beta-lactamase (EC 3.5.2.6)
2	12	Class A beta-lactamase (EC 3.5.2.6) => CARB/PSE family, carbenicillin-hydrolyzing
3	1046	Class A beta-lactamase (EC 3.5.2.6) => CTX-M family, extended-spectrum
4	3	Class A beta-lactamase (EC 3.5.2.6) => GES family
5	493	Class A beta-lactamase (EC 3.5.2.6) => KPC family, carbapenem-hydrolyzing
6	38	Class A beta-lactamase (EC 3.5.2.6) => LAP family
7	14	Class A beta-lactamase (EC 3.5.2.6) => LEN family
8	4	Class A beta-lactamase (EC 3.5.2.6) => OKP-A family, broad-spectrum
9	10	Class A beta-lactamase (EC 3.5.2.6) => OKP-B family, broad-spectrum
10	1	Class A beta-lactamase (EC 3.5.2.6) => SCO family
11	1646	Class A beta-lactamase (EC 3.5.2.6) => SHV family
12	802	Class A beta-lactamase (EC 3.5.2.6) => TEM family
13	1	Class C beta-lactamase (EC 3.5.2.6)
14	2	Class C beta-lactamase (EC 3.5.2.6) => ADC family
15	2	Class C beta-lactamase (EC 3.5.2.6) => BlaEC family
16	2	Class C beta-lactamase (EC 3.5.2.6) => DHA/MOR family
17	10	Class C beta-lactamase (EC 3.5.2.6) => FOX family, cephalosporin-hydrolyzing
18	23	Class D beta-lactamase (EC 3.5.2.6)
19	383	Class D beta-lactamase (EC 3.5.2.6) => OXA-1 family
20	6	Class D beta-lactamase (EC 3.5.2.6) => OXA-2 family
21	2	Class D beta-lactamase (EC 3.5.2.6) => OXA-24 family, carbapenem-hydrolyzing
22	8	Class D beta-lactamase (EC 3.5.2.6) => OXA-48 family, some variants carbapenem-hydrolyzing
23	2	Class D beta-lactamase (EC 3.5.2.6) => OXA-51 family, carbapenem-hydrolyzing
24	11	Subclass B1 beta-lactamase (EC 3.5.2.6) => NDM family
25	47	Aminoglycoside 2”-nucleotidyltransferase (EC 2.7.7.46) => APH(2”)-Ia (AadB family)
26	669	Aminoglycoside 3”-nucleotidyltransferase (EC 2.7.7.-) => APH(3”)-Ia (AadA family)
27	2	Aminoglycoside 3”-nucleotidyltransferase, putative
28	531	Aminoglycoside 3”-phosphotransferase (EC 2.7.1.87) => APH(3”)-I
29	345	Aminoglycoside 3’-phosphotransferase (EC 2.7.1.95) => APH(3’)-I
30	48	Aminoglycoside 3’-phosphotransferase (EC 2.7.1.95) => APH(3’)-II/APH(3’)-XV
31	8	Aminoglycoside 3’-phosphotransferase (EC 2.7.1.95) => APH(3’)-III/APH(3’)-IV/APH(3’)-VI/APH(3’)-VII
32	31	Aminoglycoside 4-phosphotransferase (EC 2.7.1.-) => APH(4)-I
33	535	Aminoglycoside 6-phosphotransferase (EC 2.7.1.72) => APH(6)-Ic/APH(6)-Id
34	1	Aminoglycoside 6-phosphotransferase, putative
35	646	Aminoglycoside N(3)-acetyltransferase (EC 2.3.1.81) => AAC(3)-II,III,IV,VI,VIII,IX,X

Column	Count	Function
36	3	Aminoglycoside N(6')-acetyltransferase (EC 2.3.1.82) => AAC(6')-Ia (and related AACs)
37	707	Aminoglycoside N(6')-acetyltransferase (EC 2.3.1.82) => AAC(6')-Ib/AAC(6')-II
38	1665	Aminoglycosides efflux system AcrAD-TolC, inner-membrane proton/drug antiporter AcrD (RND type)
39	369	Tetracycline resistance, MFS efflux pump => Tet(A)
40	5	Tetracycline resistance, MFS efflux pump => Tet(B)
41	328	Tetracycline resistance, MFS efflux pump => Tet(D)
42	3	Tetracycline resistance, MFS efflux pump => Tet(J)
43	2	Tetracycline resistance, ribosomal protection type => Tet(M)
44	1	Tetracycline resistance, ribosomal protection type => Tet(W)
45	487	Macrolide 2'-phosphotransferase => Mph(A) family
46	20	Macrolide 2'-phosphotransferase => Mph(E)/Mph(G) family
47	1666	Macrolide export ATP-binding/permease protein MacB
48	17	Macrolide resistance, MFS efflux pump => Mef(B)
49	1666	Macrolide-specific efflux protein MacA
50	322	Chloramphenicol O-acetyltransferase (EC 2.3.1.28) => CatA1/CatA4 family
51	217	Chloramphenicol O-acetyltransferase (EC 2.3.1.28) => CatA2 family
52	388	Chloramphenicol O-acetyltransferase (EC 2.3.1.28) => CatB family
53	56	Chloramphenicol resistance, MFS efflux pump => CmlA family
54	20	Chloramphenicol/florfenicol resistance, MFS efflux pump => FloR family
55	1663	AcrZ membrane protein associated with AcrAB-TolC multidrug efflux pump
56	1666	Multidrug efflux system AcrAB-TolC, inner-membrane proton/drug antiporter AcrB (RND type)
57	1666	Multidrug efflux system AcrAB-TolC, membrane fusion component AcrA
58	1666	Multidrug efflux system AcrEF-TolC, inner-membrane proton/drug antiporter AcrF (RND type)
59	1666	Multidrug efflux system AcrEF-TolC, membrane fusion component AcrE
60	1663	Multidrug efflux system EmrAB-OMF, inner-membrane proton/drug antiporter EmrB (MFS type)
61	1663	Multidrug efflux system EmrAB-OMF, membrane fusion component EmrA
62	13	Multidrug efflux system EmrKY-TolC, inner-membrane proton/drug antiporter EmrY (MFS type)
63	4	Multidrug efflux system EmrKY-TolC, membrane fusion component EmrK
64	1665	Multidrug efflux system MdtABC-TolC, inner-membrane proton/drug antiporter MdtB (RND type)
65	1665	Multidrug efflux system MdtABC-TolC, inner-membrane proton/drug antiporter MdtC (RND type)
66	1665	Multidrug efflux system MdtABC-TolC, membrane fusion component MdtA
67	4	Multidrug efflux system MdtEF-TolC, inner-membrane proton/drug antiporter MdtF (RND type)
68	4	Multidrug efflux system MdtEF-TolC, membrane fusion component MdtE
69	2	Multidrug efflux system, inner membrane proton/drug antiporter (RND type) => MexB of MexAB-OprM
70	1	Multidrug efflux system, inner membrane proton/drug antiporter (RND type) => MexD of MexCD-OprJ system

Column	Count	Function
71	5	Multidrug efflux system, inner membrane proton/drug antiporter (RND type) => MexI of MexHI-OpmD system
72	6	Multidrug efflux system, inner membrane proton/drug antiporter (RND type) => MexQ of MexPQ-OpmE system
73	1	Multidrug efflux system, membrane fusion component => MexC of MexCD-OprJ system
74	3	Multidrug efflux system, membrane fusion component => MexH of MexHI-OpmD system
75	6	Multidrug efflux system, membrane fusion component => MexP of MexPQ-OpmE system
76	1	Multidrug efflux system, outer membrane factor lipoprotein => OprJ of MexCD-OprJ system
77	3	Multidrug efflux system, outer membrane factor lipoprotein of OprM/OprM family
78	4	Outer membrane factor (OMF) lipoprotein associated wth EmrAB-OMF efflux system
79	7	23S rRNA (adenine(2058)-N(6))-dimethyltransferase (EC 2.1.1.184) => Erm(B)
80	2	3-hydroxyacyl-CoA dehydrogenase (EC 1.1.1.35), FabG4
81	20	ABC-F type ribosomal protection protein => Msr(E)
82	1667	Carbohydrate-selective porin OprB
83	1657	Catalase-peroxidase KatG (EC 1.11.1.21)
84	13	Erythromycin esterase (EC 3.1.1.-) => EreA family
85	1666	Hydrogen peroxide-inducible genes activator => OxyR
86	1660	Multidrug resistance regulator EmrR (MprA)
87	1665	Multidrug resistance transporter => Bicyclomycin resistance protein Bcr
88	1660	Multiple antibiotic resistance protein MarA
89	1659	Multiple antibiotic resistance protein MarB
90	1660	Multiple antibiotic resistance protein MarR
91	1667	Outer membrane channel TolC (OpmH)
92	3	Outer membrane low permeability porin, OprD family
93	1	Outer membrane low permeability porin, OprD family => OccD2/OpdC
94	1664	Outer membrane low permeability porin, OprD family => OccD6/OprQ involved in adhesion
95	1	Pellicle/biofilm biosynthesis inner membrane protein PslK, MATE transporter family
96	1	Pellicle/biofilm biosynthesis protein PslA, polyprenyl glycosylphosphotransferase
97	78	Pentapeptide repeat protein QnrB family
98	281	Pentapeptide repeat protein QnrB family => Quinolone resistance protein QnrB10
99	2	Probable (3R)-hydroxyacyl-CoA dehydratase HtdX
100	3	Transcriptional activator GadE
101	1665	Transcriptional regulator of acrAB operon, AcrR
102	1	Two component system sensor histidine kinase MtrB

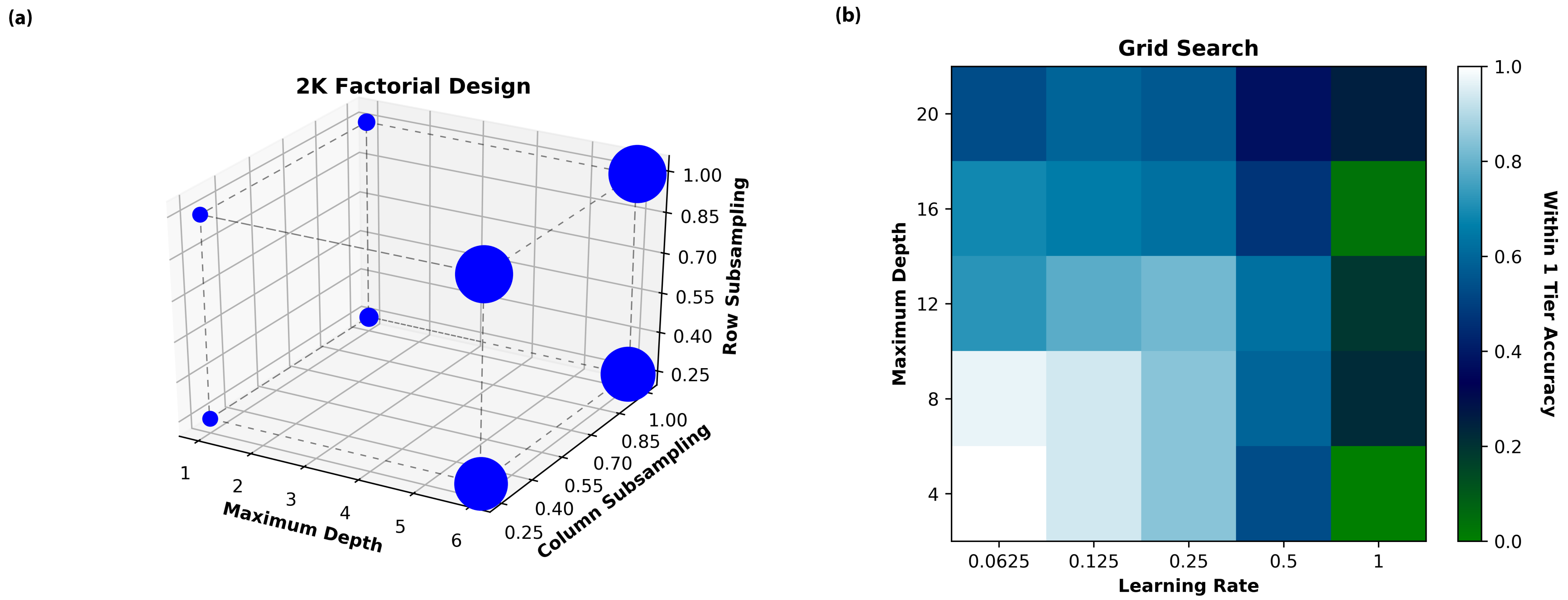

Figure S1. Results of the 2^k factorial design on the XGBoost model. **(a)** Three dimensional plot showing the relationship between maximum depth, column subsampling and row subsampling parameters. The size of the spheres represent the within 1-tier accuracy score for the given model. Larger spheres indicate higher accuracy. Dashed lines are added to aid in visualization. The image shows that maximum tree depth plays the largest role in the XGBoost model with column and row subsampling having smaller roles. **(b)** Heat map showing the relationship between learning rate and maximum tree depth. In the color scheme, white is most accurate and dark blue and green are least accurate. The image shows that lower depth and learning rates produce more accurate models.

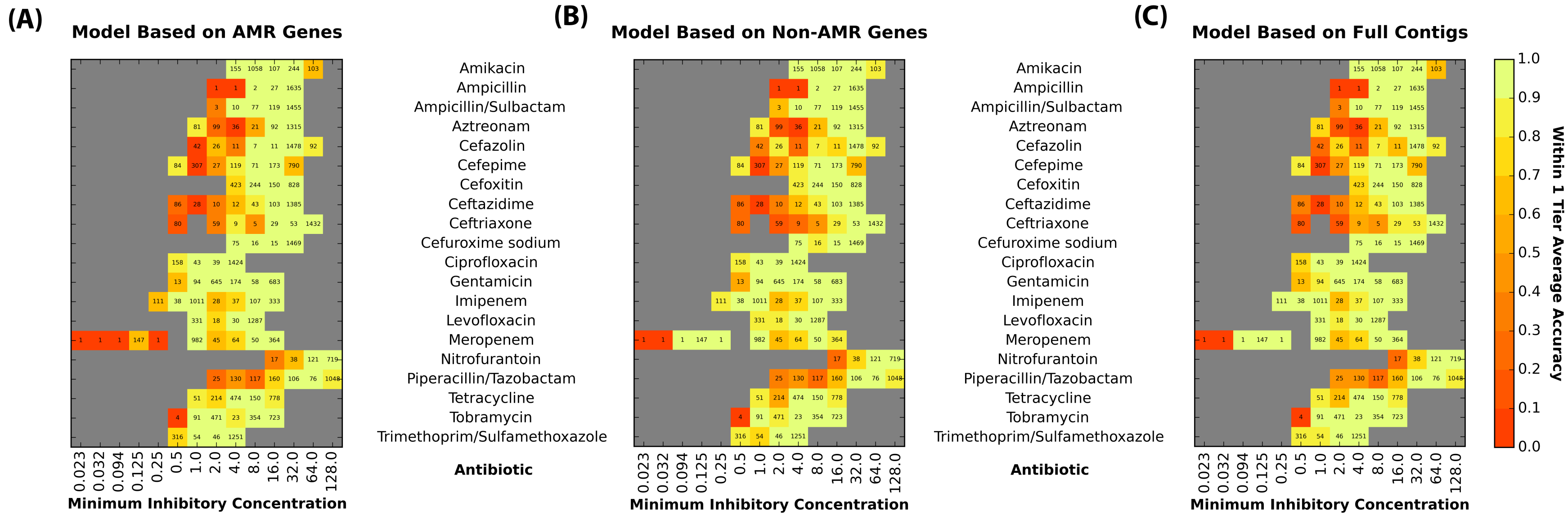

Figure S2. Heat maps comparing the accuracies of the XGBoost model for individual MICs generated from (A) AMR genes, (B) Non-AMR genes, and (C) full contigs. The X-axis of the heatmap shows shows the actual MIC (μg/ml) for a bin and the Y-axis lists the antibiotics. The within $\pm$1-tier accuracy of a particular antibiotic-MIC bin is denoted by color, with red and orange being least accurate and bright yellow and green being most accurate. The number within each cell represents the number of samples (genomes with the MIC) within the bin. The data depict genomes for which there is at least one AMR gene called by PATRIC or CARD.

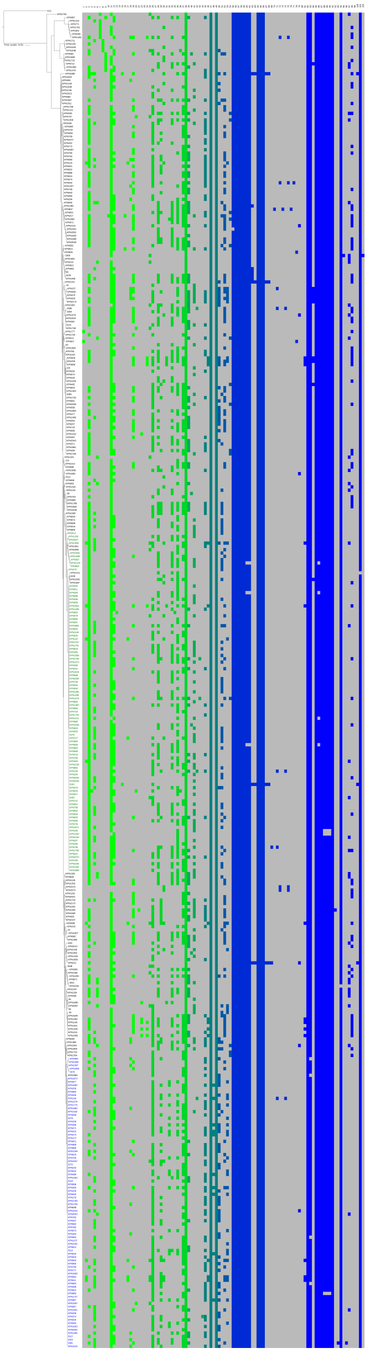

Figure S3. A phylogenetic tree of *K. pneumoniae* isolates used in this study and the distribution of AMR genes within each genome. The tree is based on a concatenated alignment of the MLST protein encoding genes and is rooted on *E. coli* K-12 substr. MG1655 (K12). Genomes belonging to MLST 307 and 258 are are colored green and blue respectively. 409 of the original 1668 genomes with differing sets of AMR genes are shown. Each column in the heatmap depicts the absence (gray cells) or presence (cells with other colors) of genes with AMR functions based on either PATRIC or CARD. Columns 1-24 represent genes with functions relating to beta-lactam resistance (bright green), columns 25-38 represent genes with functions relating to aminoglycoside resistance (green), columns 39-44 represent genes with functions relating to tetracycline resistance (dark green), columns 45-49 represent genes with functions relating to macrolide resistance (blue green), columns 50-54 represent genes with functions relating to chloramphenicol resistance (gray-blue), columns 55-78 represent genes with functions relating to efflux mechanisms (blue), and columns 79-102 represent genes with other AMR-related functions (bright blue). The functions for each column of the heat map are shown in Table S7. Strain names with the prefix KPN were described previously[3], numbered strains are new to this study.
